# The ninth art, tells the story of cultural China and the world: Cultural interaction in cross-cultural video game communication-based on ’Genshin Impact’ YouTube comments

**DOI:** 10.1371/journal.pone.0311317

**Published:** 2024-10-25

**Authors:** Bo Zhang, Mengyue Zhou, Chuyuan Pu

**Affiliations:** College of Publishing, University of Shanghai for Science and Technology, Shanghai, China; Kitami Institute of Technology, JAPAN

## Abstract

China has attached increasing importance to the overseas dissemination of traditional culture as it continues to strengthen its comprehensive national strength and develop globalization initiatives. The electronic game, as the ’ninth art’, with its booming development and inherent cross-cultural communication platform attributes, has taken on the important task of taking culture abroad. This study selects ’Genshin Impact’, a game highly praised and well-received globally, which contains an abundance of Chinese cultural elements and cultural elements from various regions of the world, to study cultural interaction in cross-cultural communication of electronic games. This study conducts text sentiment analysis and semantic network analysis on the comments of ’Genshin Impact’ videos on the overseas video platform YouTube and studies the cultural interaction in the cross-cultural communication of the ’ninth art’, telling the cultural stories of China and the world from a multicultural perspective. The study found that games and derivative videos triggered positive emotional responses from audiences both at home and abroad. While experiencing pleasure and surprise, audiences also arouse deeper emotions such as admiration, identification, and expectation. Meanwhile, in the complex art form of the ’ninth art’, audiences can absorb the cultural elements of different cultures, resonate with the elements of their cultural circle, and even trigger further profound thinking. Finally, this study provides constructive suggestions on using the ’ninth art’ to better ’tell China’s stories and disseminate China’s voice’.

## 1. Introduction

Current studies on video games and cross-cultural communication mostly employ qualitative research methods, focusing on the subjects of the study and communication strategies. Moreover, in the research on ’Genshin Impact’ and cultural communication, the focus is only on the export of traditional Chinese culture, both domestically and globally.

There are studies based on the case of ’Genshin Impact’, providing insights for promoting the cross-border cooperation of the game industry, the innovative model of China’s cultural tourism industry, and sustainable development [1]. Hu Yu et al. and Wang Peinan [2, 3] also discussed innovative models of the external communication of traditional Chinese culture via online games, particularly overseas communication, and the transformation and upgrading of global communication on Chinese Internet platforms, using ’Genshin Impact’ as an example. Few studies have investigated cultural interaction in the cross-cultural communication of video games through direct access to authentic and objective audience data.

The innovation of this study lies in retrieving comments from the ’Genshin Impact’ video comment section on the overseas video website YouTube and conducting an online text analysis of a large amount of the most authentic and objective data to investigate cultural interaction in the cross-cultural communication of video games.

The study identified the languages of the collected comments, with 75 in total. After translating them into English, an emotional analysis was performed to understand overseas audiences’ emotional polarity and experiences with ’Genshin Impact’ and its derivative videos. In addition, semantic network analysis was conducted to investigate cultural interaction in the cross-cultural communication of ’Genshin Impact’ and its derivative videos from a multicultural perspective. This study examines the effectiveness and outcome of ’Genshin Impact’ as the ’ninth art form’ in telling cultural stories of China and the world from the audience’s perspective in various countries and regions worldwide.

The comprehensive research findings show that ’Genshin Impact’ and its derivative videos not only provide a positive emotional experience and emotional value to audiences but also successfully tell the cultural stories of China and the world through its immersive qualities as a masterpiece of the ’ninth art form’, even stimulating further reflection among audiences. Finally, the study provides policy implications for using the ’ninth art form’ to ’tell Chinese stories well and spread good Chinese voices’.

## 2. The ’Ninth art’ and Genshin impact

The Ninth Art refers to a form of art transcending the eight traditional major art forms (painting, sculpture, architecture, music, literature, dance, drama, and film). The definitions of ninth art vary across countries and sources, such as television art, comics, and video games. In 1997, Chinese scholar Wu Guanjun referred to video games as the ninth art that became popular in China. Video games can be called the ninth art because they inherit the expressiveness of films as the eighth art while also having their unique interactivity and creativity [4]. Video games can showcase visuals, music, plots, and other elements and allow players to participate in the game world and experience different roles and emotions. The way games express and narrate stories is something that other art media cannot achieve, making it a unique, expressive form of video games.

Genshin Impact is an open-world action role-playing game developed and published by Chinese game developer miHoYo. The game is set in a continent called ’Teyvat’, where players can learn about the game’s lore by following the main storyline and strengthening character cognition and game story experience through legendary quests and invite events [5]. In addition, Genshin Impact features hidden quests and secrets that players can discover and solve. The game’s graphics and sound effects are outstanding, allowing players to experience rich and colorful scenes and visual effects, immersive, and feel the various atmospheres in the game. It is well-loved by players worldwide and can be considered as a masterpiece of ’ninth art’ from China.

Genshin Impact has garnered high attention and acclaim globally, becoming one of the most-watched games in recent years. It has excellent search popularity on Google worldwide, especially in the Philippines, Japan, the United States, and other countries. Since its launch in 2020, Genshin Impact has achieved tremendous domestic and international success. It ranked first on overseas revenue charts for 18 consecutive months and is still the leading Chinese mobile game overseas in revenue and growth. According to Sensor Tower, in 2022, miHoYo’s Genshin Impact clinched the crown of China’s top overseas mobile game through annual revenue and its continuous release of premium content updates. It made the 2022 top-grossing mobile game charts in the US and Japan, the first and second-largest overseas markets for Chinese mobile games. In January 2023, Genshin Impact’s mobile revenue was $150 million, of which 41.2% came from China’s iOS market, 23.7% from Japan, and 10.2% from the US [6].

In summary, the Genshin Impact game is an open-world ’ninth art’ masterpiece that provides an excellent immersive experience, and its outstanding performance and widespread attention globally also pave the way for spreading stories and cultural elements within. At the same time, Genshin Impact and its derivative videos intricately incorporated cultural symbols from ancient China, Japan, Europe, and other countries and regions. Therefore, the following research questions were proposed:

Question 1: What sentiment polarities do overseas players and viewers show towards overseas dissemination of Genshin Impact and derivative videos?
Question 2: What kinds of sentimental experiences do overseas players and viewers have towards the Chinese and world cultures presented in the dissemination of Genshin Impact and its derivative videos?
Question 3: What content is shown in topics related to Genshin Impact and its derivative videos?
Question 4: Do overseas players emotionally and culturally resonate with world stories and cultural elements in Genshin Impact and its derivative videos?
Question 5: How can we better conduct cross-cultural communication with the ’ninth art’ to ’tell Chinese stories well and spread Chinese voices’?

## 3. Literature review

### (1) Culture and games

Video games are cultural products and human-made cultural or cognitive artifacts. At the same time, because of their nature as mass media, they have enormous social importance [7]. Chen adopted a quantitative and qualitative approach to explore the cultural power of video games from the cultural imperialism theory perspective, affirming the role of video games as cultural carriers [8].

Thus, research on culture and games has become an important academic field. As a cultural medium, games involve many social, cultural, cognitive, and interactive issues, in addition to entertainment. Corliss stated that ’all games can be analyzed as sociocultural phenomena’ [9]. Bourdieu viewed video game production as a cultural field [10]. Brendan Keogh followed Bourdieu’s perspective and combined the local context in Australia, regarding game creators as cultural producers, and discussing the significance of seriously treating video game production as a cultural production field [11]. At the same time, some argue that, like all cultural workers, video game creators ’exist at the nexus of political struggles between artistic and commercial forces’ [12]. That is to say, the game works created by game creators are not just entertainment products; like other cultural works, they also carry social, cultural, and political meanings.

A close connection exists between culture and games. As a form of cultural expression, games carry cultural elements such as social, historical, and values. By studying cultural symbols, values, and communication in games, we can understand the mutual influence between games and culture. Video games can subtly influence players’ cognition by carrying cultural symbols in immersive game experiences. They can become new carriers of traditional cultural content in the digital age [13]. Therefore, scholars in the field of cultural communication should pay more attention to the role of games as cultural carriers, and study the effects of cultural symbols spreading along with games. To investigate the cultural communication effects of games, big data methods should be combined to obtain the real and intuitive reactions of recipients in different or similar cultural circles, such as comments on game derivative videos or game community forums and discussion posts, and conduct semantic analysis, sentiment analysis, etc., to more comprehensively and thoroughly examine the cultural interaction and cross-cultural communication effects.

### (2) Cultural interaction and output in games

The theory of cultural interaction is one of the most unique and creative academic thoughts of Russian cultural symbologist Yuri Lotman, who enjoys an international reputation. It provides a theoretical basis for analyzing cultural communication and collisions [14]. It includes the following aspects: theory of semiospheres, theory of cultural texts, modes of text communication, and dynamic mechanisms of cultural dialogues. Among them, the theory of cultural texts refers to texts in cultural symbolic systems, including language, images, and music, which are important carriers for people to conduct cultural exchanges [15]. Stories, characters, scenes, music, and games are cultural texts that can reflect the themes, styles, values, and cultural symbols contained within games. In the Genshin Impact game, the costume design of Zhongli, the Geo Archon in the Liyue (ancient China) region, is closely related to the dragon robes of ancient Chinese emperors, such as the frontal clasps and dragon scale design at the end of the costume, which incorporates China’s dragon totem culture. Other exotic cultural symbols, such as the omikuji fortune slips and fortune-telling by a shrine maiden at the Grand Narukami Shrine in the game showcase Japan’s shrine culture.

Games are cross-cultural media that can interact with and influence other cultural systems. Scholars, such as Garcia-Fernandez, have evaluated the ability of the electronic game Minecraft as an effective tool for conveying architectural heritage environments, considering the determinants of immersion, motivation, and simulation fidelity, and exploring the role of electronic games in cultural heritage communication and cultural output [16]. However, regarding Chinese cultural outcomes, relying solely on cultural outputs is insufficient. To achieve better cultural output results, allowing ’others’ to actively understand and recognize Chinese culture is necessary. Mutual cultural interaction and understanding promote cultural transmission and mutual respect [17].

It is worth noting that game text is an open, dynamic, and polysemous symbolic expression form. It requires active participation, interpretation, and interaction from players to generate meaning and experience, which is conducive to cultural interaction. David Morley believes that in the close interaction of transnational geography, cultural subjects must not only look at the world from the perspective of ’others’, but also let ’others’ come to ’find’ us, ’influence’ us, and even ’deny’ us [18]. As ’others’, foreign players come to ’find’ Chinese culture in Chinese games, which is exactly the essence of Chinese cultural output.

Cultural interaction theory studies human interactive behavior in social and cultural contexts. Cultural interaction theory can be used to explore the role of games in player social interaction and cultural exchange. Researchers can focus on issues such as social interactions in games, the construction of cultural identity, cross-cultural exchange, and the impact of games on individual and group cultural cognition. As can be seen, electronic games have great potential in cultural communication and cultural output, and the further expansion of the current game market scale creates considerable demand and space for research and development in academia and industry.

## 4. Research methods and analysis process

### (1) Data source

The data for this study were obtained from video comment sections on the Genshin Impact official YouTube account.

First, veteran Genshin Impact enthusiasts and experts selected high-quality videos that contained abundant cultural elements from regions worldwide. They divided the videos into ’Cultural China’,’ World Stories’, and Real-World Stories based on the video content for comment scraping. Simultaneously, to ensure the timeliness of the comment content, this study only scraped the latest approximately 1000 comments in the comment sections of the selected videos up to May 2023. Finally, the ’Cultural China’ section obtained 12,684 valid comments, and the ’World Stories’ section obtained 9,257 valid comments, totaling 21,941 valid YouTube comments. To facilitate subsequent analysis and research, the comment data were batch-translated into English via the Baidu Translation API, and the language types were statistically analyzed. Part of the translation results and language-type records are shown in Table 1.

**Table 1:** Part of the translation results and language types.

Number	Comment	Original Language
1	This trailer is still my favorite.	Spanish
2	How many memories.	Portuguese
3	I am willing to review it again and again.	Russian
4	I will be waiting for the mail to come back to me today.	Japanese
5	woww	French
6	The Great Shogunate General	Thai
7	I can’t stay for a moment with this 3.0	Chinese
8	Good thing they posted this. I’m not able to view this on my phone, only the audio played in-game.	English
9	Snow as fast as he can.	Afrikaans
10	Nilo’s love.	Farsi

### (2) Text sentiment analysis

#### ①. Sentiment polarity analysis

For the text processing of YouTube comments, this study adopted the TextBlob package, which provides various functions, including word segmentation, part-of-speech tagging, sentiment analysis, and translation. Because YouTube comments cover multiple languages, the comment data translated by the Baidu Translation API were used as the data source for research.

The sentiment method in TextBlob can score the sentiment polarity of text. The scoring range of sentiment polarity is [–1, 1], where -1 represents completely negative and 1 represents completely positive. The closer the value is to 1, the more positive the sentiment the comment contains; the closer the value is to -1, the more negative the sentence. This feature helps us to understand the sentiment tendencies of comments.

#### ②. Sentiment experience type analysis

For YouTube comments, the sentiment analysis method used in this study is based on the NRC lexicon (including various discrete emotions). The NRC lexicon was created by experts from the National Research Council of Canada (NRC) and currently has versions in multiple languages. It lists English words and their associations with eight basic emotions (anger, fear, anticipation, trust, surprise, sadness, joy, and disgust) and two emotions (negative and positive). Negative and positive sentiments have already been analyzed in sentiment polarity analysis; therefore, they are not repeated in this step. In addition to the eight basic emotions (anger, fear, anticipation, trust, surprise, sadness, joy, and disgust) already included in the NRC lexicon, the emotion vocabulary ’admire’ was manually added based on the topic of these YouTube comments, as words like ’awesome’, ’goosebumps’, ’admire’ admire, which appear frequently in the comments and express netizens’ appreciation, admiration, and praise for the Genshin Impact game.

Similarly, sentiment experience type analysis was performed on 21,941 English comments on YouTube. The comment data were segmented into individual words by space and processed as stop words. After preliminary screening of the vocabulary, based on the improved NRC emotional vocabulary, its emotional vocabulary system is divided into nine major emotions ’anger’, ’anticipation’, ’disgust’, ’fear’, ’joy’, ’sadness’, ’surprise’, ’admire’ and ’trust’. Among them, 241 comments in the ’Cultural China’ category and 173 comments in the ’World Stories’ category do not contain emotional vocabulary and cannot be labeled with sentiment categories. These comments were manually corrected to identify and add emotional vocabulary to the NRC’s emotional vocabulary system. Simultaneously, the new sentiment category ’admire’ was added, and words like ’admire’ and ’goosebumps’ were classified into this sentiment category. The improved sentiment dictionary contained nine major categories and 14,225 English emotional vocabularies (see Table 2 below).

**Table 2:** The nine English emotion classification systems.

Number	Emotional Category	Example Words
1	anger	abandoned, betray, deceit
2	anticipation	delight, elegance, hopeful
3	disgust	hideous, nauseous, plagiarism
4	fear	poison, scar, sorrow, steal
5	joy	successful, volunteer, winner
6	sadness	violently, overwhelmed, refugee
7	surprise	reflex, secrecy, stealthy, urgency
8	admire	love, awesome, goosebumps, admire
9	trust	edification, governess, peace

### (3) Semantic network analysis

To present the relationship between sentiment experiences and sentiment objects more clearly, this study utilizes semantic network analysis to conduct an in-depth analysis of users’ key emotional characteristics and main emotional objects.

#### ①. Word frequency analysis

After translating the English comments, stop words were removed, and nouns, adjectives, and verbs were retained for word frequency statistics to create word cloud diagrams for analysis and to more intuitively understand where the discussion focuses on players and video viewers.

#### ②. Co-word analysis

To effectively identify the key emotional objects in user comments, this study calculated the centrality of nodes in the co-word network because the centrality of nodes in the co-word network can be used to measure the importance of a keyword in the network. The key indicators for measuring node centrality are degree, betweenness, and closeness centralities. Degree centrality can be used to measure the keywords that are the most important in the co-word network. The larger the node degree, the higher is the degree of centrality. Betweenness centrality refers to the number of shortest paths that pass through a node. The closeness centrality reflects the closeness between a node and other nodes, and nodes with higher values are more core. The specific steps are as follows.

First, we used the keywords of the top 50 frequent words in the high-frequency words. According to the characteristics of English words, some repetitive verbs such as cry, crying, and crying merged into crying. Python was used to tag and count the number of times the 50 keywords appeared in the YouTube comment section. Afterward, 50 × 50 co-occurrence matrices of keywords were established for ’Cultural China’,’ World Stories’, and Real-World Stories, respectively (see Tables 3 and 4). Next, the keyword co-occurrence matrices were input into the social network analysis software Ucinet 6.4 and converted into binary matrices to calculate the centrality of the nodes in the co-word network. Finally, Gephi 0.9.2 was used to draw co-occurrence graphs of high-frequency words. The font sizes of the nodes and labels were set during drawing, according to the node degree value. A larger font indicates more co-occurrences of a node with other nodes. The thickness of the edges was also selected according to the node degree, with thicker borders indicating more co-occurrence between the two nodes. This method can help visualize co-occurrence relationships to better understand the connections between keywords.

**Table 3:** Co-occurrence matrix of key terms in ’Cultural China’ reviews.

	amazing	art	beautiful	character	Chinese	cloud	crying	culture	cute
**amazing**	78	36	61	59	35	7	19	27	10
**art**	36	28	65	16	28	1	3	36	1
**beautiful**	61	65	130	98	116	12	86	60	6
**character**	59	16	98	406	45	32	6	7	22
**Chinese**	35	28	116	45	336	6	26	177	2
**cloud**	7	1	12	32	6	28	8	0	4
**crying**	19	3	86	6	26	8	38	5	8
**culture**	27	36	60	7	177	0	5	46	0
**cute**	10	1	6	22	2	4	8	0	12

**Table 4:** Co-occurrence matrix of keywords in comments on ’A Story of the World’.

	amazing	anniversary	archon	beautiful	character	Chen	cool	cutscene	dance
**amazing**	92	24	25	39	89	21	18	8	8
**anniversary**	324	36	0	5	9	12	2	0	0
**archon**	25	0	100	23	32	17	1	9	12
**beautiful**	39	5	23	36	63	7	12	26	28
**character**	89	9	32	63	224	5	27	10	2
**Chen**	21	12	17	7	5	6	1	0	1
**cool**	18	2	1	12	27	1	12	2	0
**cutscene**	8	0	9	26	10	0	2	34	14
**dance**	8	0	12	28	2	1	0	14	18

#### ③. Cluster analysis

By applying block model analysis in social network analysis, the keyword co-occurrence matrix was imported into Ucinet 6.4 to calculate the density of keyword subgroups. Subsequently, dendrograms and block models were created to obtain cohesive subgroups of keywords. This analysis can help judge the audience’s major emotional objects and themes overall, thereby better understanding the discussion focal points in disseminating Genshin Impact and the derived videos. This is a powerful method that can help gain an in-depth understanding of audience concerns.

## 5. Research results

### (1) Data representativeness: Diverse perspectives under multilingual conditions

This study used the Baidu translation API to identify languages from 21,942 valid comments obtained from YouTube. It translated non-English texts into English using general text translations to facilitate subsequent analyses.

A total of 74 languages were identified (Table 5), with English being the most prevalent, followed by Russian, Spanish, Chinese, Portuguese, and Indonesian. The range of languages used spans the world on five continents, which is associated with the global nature of YouTube video platforms. The dominance of English is directly linked to its status as an international language, which does not imply a lack of richness among users from diverse countries in the comments section. As can be seen, the game ’Genshin Impact’ has a wide range of focus and spread beyond major foreign markets such as Asia and Southeast Asia, and includes regions such as Iceland and Norway in Northern Europe as well as African countries with relatively lower entertainment, economic, and game hardware development rates. The diversity and richness of languages in the comment section represent the data, and the diverse perspectives are sufficient for analyzing the cultural interaction of ’Genshin Impact’ and its derivative videos in cross-cultural transmission as the ’Ninth Art’.

**Table 5:** Distribution and quantity of languages in comment section.

English	19045	Polish	24	Dutch	7	Hindi	2
Russian	763	Croatian	23	Kurdish	6	Sinhalese	2
Spanish	560	Czech	23	Syfriac	6	Esperanto	2
Chinese	273	Albanian	23	Korean	6	Gujarati	2
Portuguese	186	Macedonian	19	Tagalog	5	Marathi	2
Indonesian	160	Estonian	17	Latvian	4	Persian	2
Italian	81	Japanese	16	Armenian	4	Tajikc	1
Vietnamese	70	Berber	15	Kazakh	4	Nepali	1
German	59	Bosnian	13	Icelandic	4	Burmese	1
Ukrainian	47	Xhosa	12	Lithuanian	4	Irish	1
French	41	Slovenian	11	Lowland German	3	Byelorussian	1
Slovak	40	Serbian	11	Basque	3	Occitan	1
Turkish	35	Catalan	10	Breton	3	Latin	1
Thai	34	Danish	9	Greek	4	Austronesian	1
Bulgarian	34	Finnish	9	Kabyle	3		
Galician	32	Hungarian	8	Afrikaans	2		
Malay	29	Georgian	8	Swedish	2		
Arabic	29	Kiswahili	8	Malagasy	2		
Romanian	28	Norwegian	8	Welsh	2		
Asturian	25	Maltese	7	Uzbek	2		

### (2) Emotional polarity: Mainly positive emotions, with a small amount of negative emotions

To investigate how ’Genshin Impact’ communicates ’Cultural China’ and ’World Stories’, this research separately collected comments from video segments with obvious ’Cultural China’ and ’World Stories’ in the process of data acquisition. Among them, ’Cultural China’ obtained 12,684 valid comments, and ’World Stories’ obtained 9,257.

**Fig 1:**
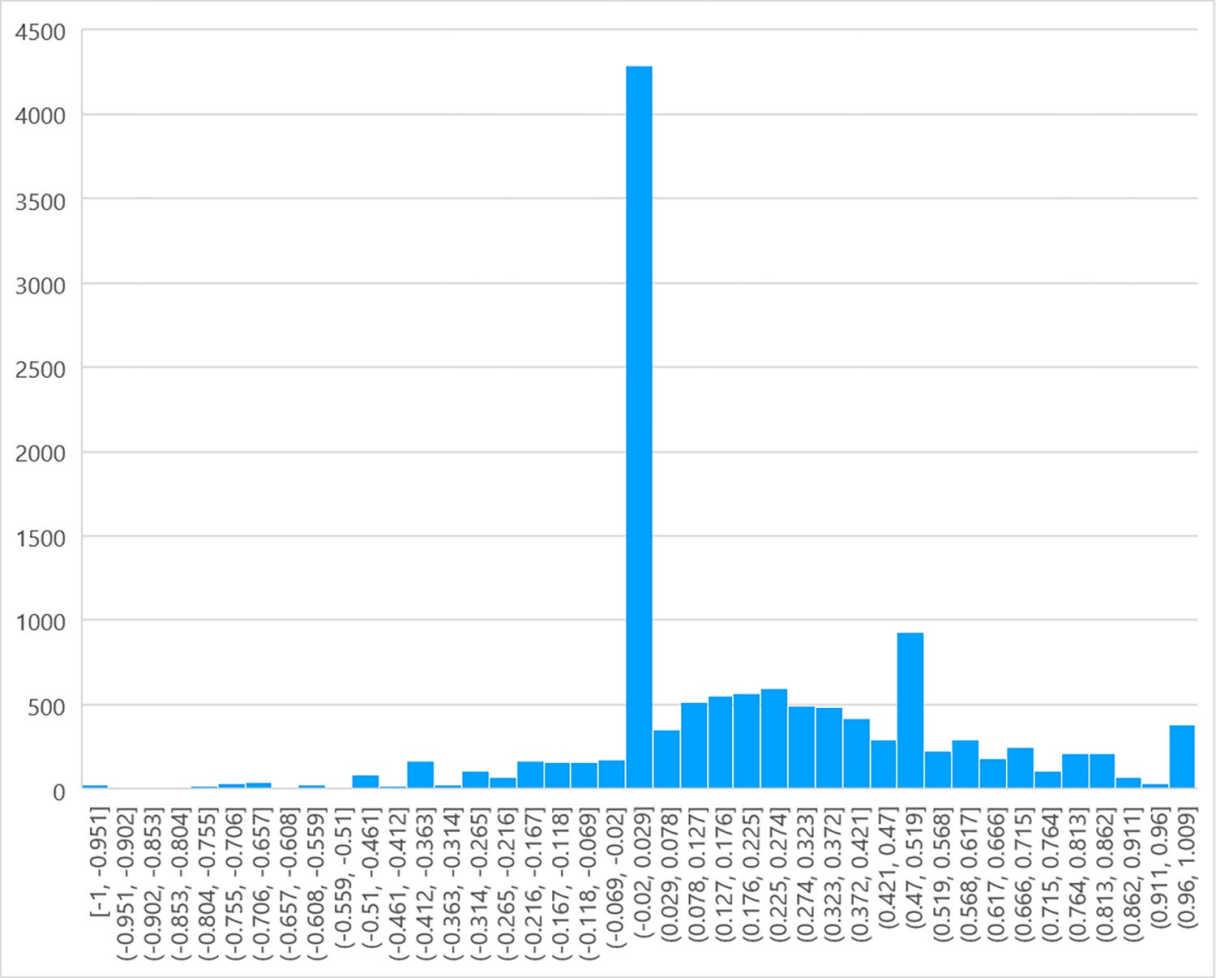
Sentiment distribution in comments section with bias towards ’Cultural China’.

**Fig 2:**
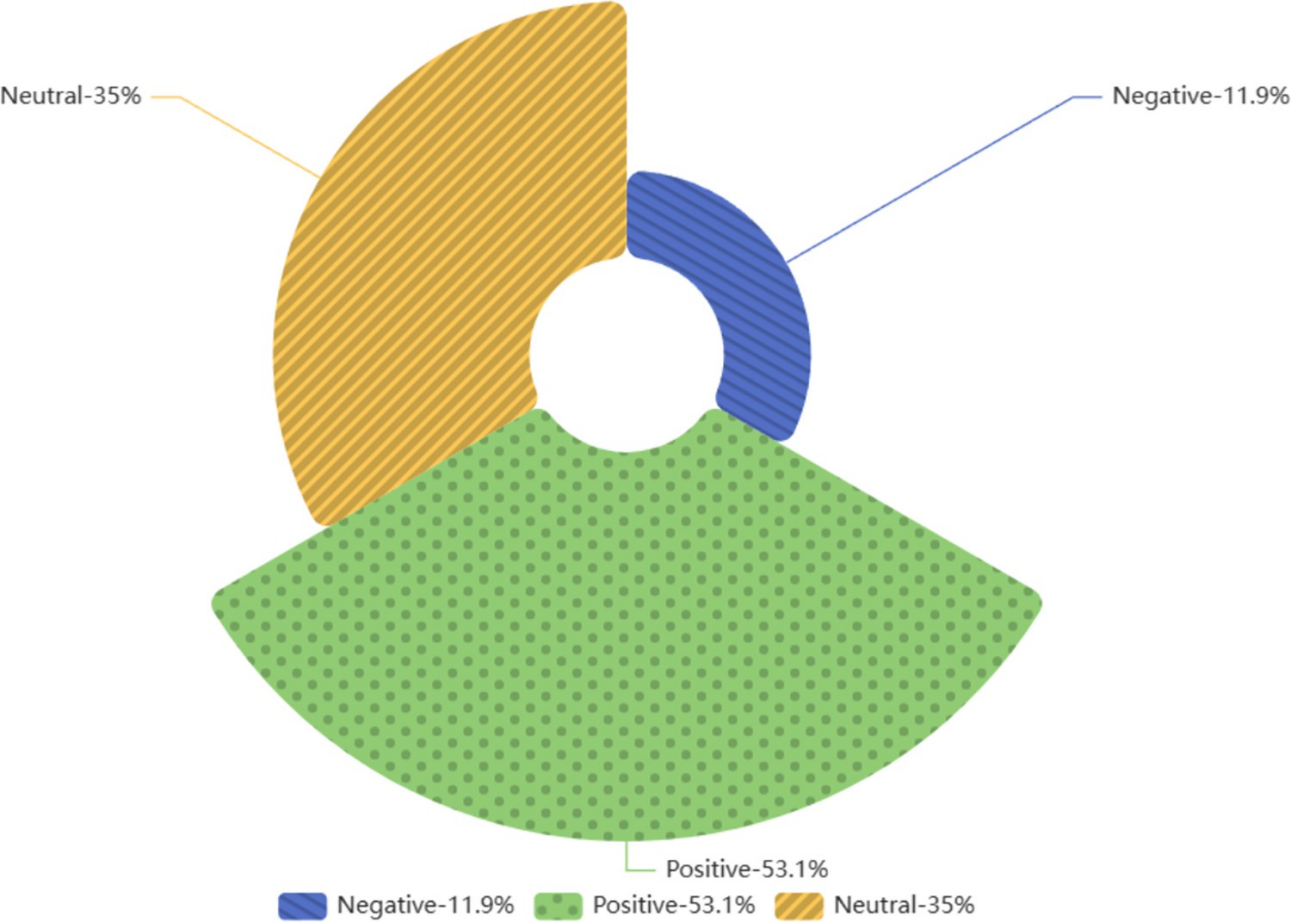
Sentiment proportion in comments section with bias towards ’Cultural China’.

As illustrated in Fig 1, a distribution graph of the sentiment scores of 12,684 YouTube comments for the ’Culture China’ section was created, with a range of [–1, –0] representing negative sentiments and the (0,1] range representing positive sentiments. The graph shows that sentiments lean towards the positive overall, with the highest concentration being mildly positive. Among the YouTube comments for the ’Culture China’ videos (see Fig 2), the percentage of positive comments was 56.8%, while negative comments made up only 10.4%, a significant difference. From these data, it can be seen that the majority of people expressed positive sentiments towards the ’Culture China’ series, indicating that this segment has won most people’s approval.

However, owing to the limitations of the recognition algorithm’s precision, sentences that contain negative emotional words but express positive or complex emotions may be misidentified as negative comments. For example, ’As a Chinese, this story had somehow really hit the spot with a weird sense of nostalgia…needless to say, I’m thankful for this festival that was rooted from tradition mixed in with a bit of mythical legend from the game itself (despite there being no Genshin Impact counterpart for mooncakes, and yes, this somewhat disappointed me)’. Therefore, compared to existing data, the actual number of positive comments should be higher.

**Fig 3:**
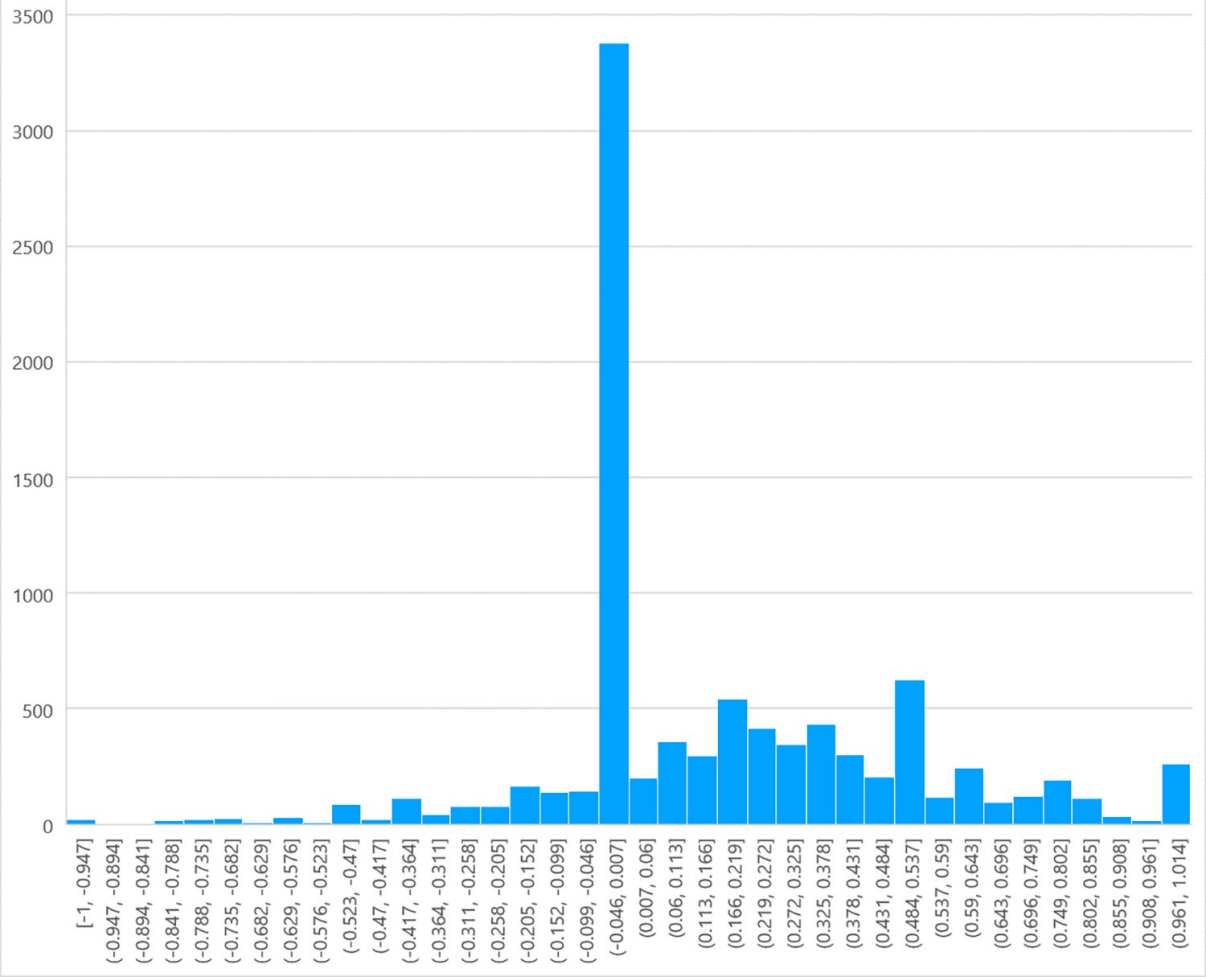
Sentiment distribution in comments section with bias towards ’A Story of the World’.

**Fig 4:**
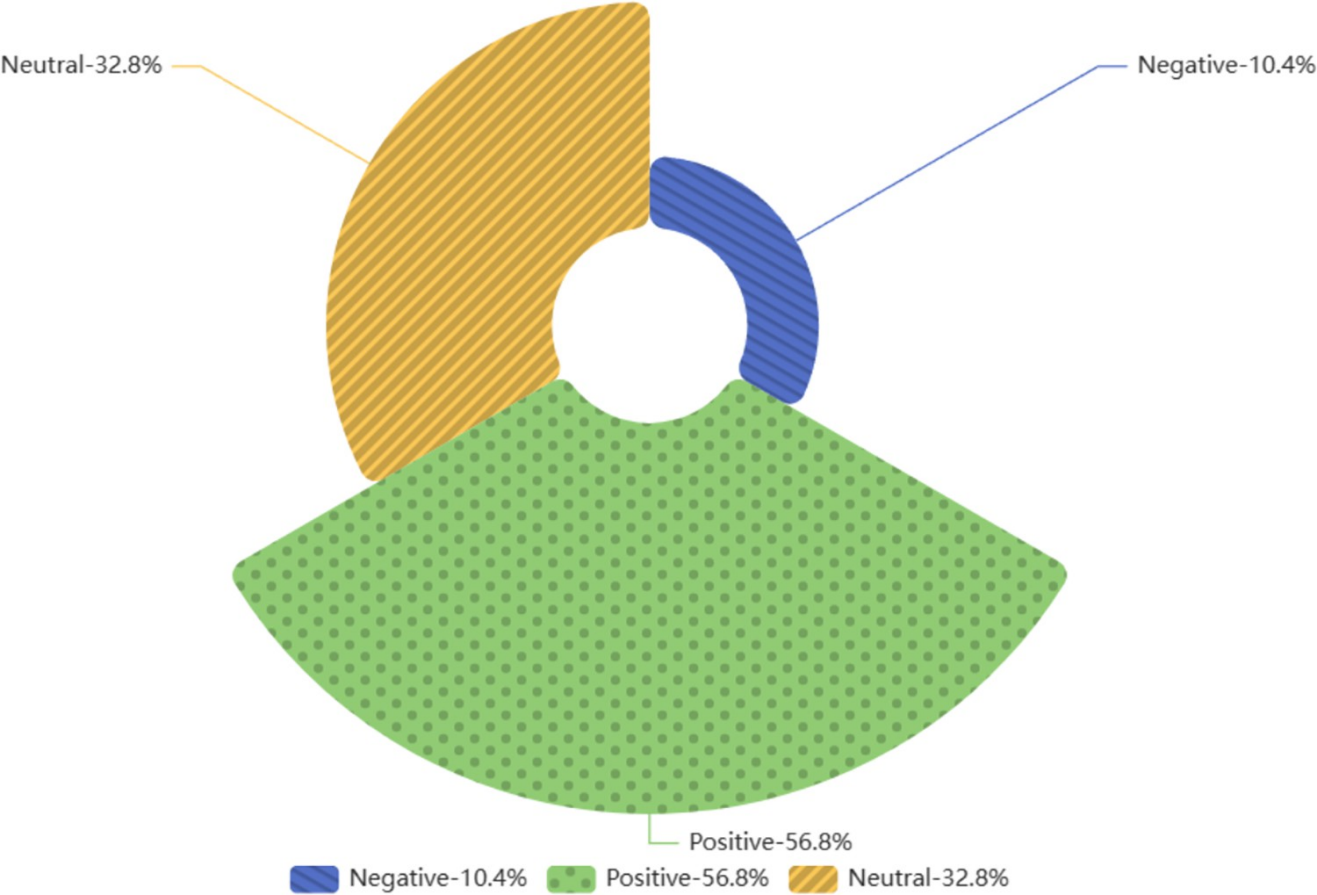
Sentiment proportion in comments section with bias towards ’A Story of the World’.

The sentiment distribution of 9,257 YouTube comments on the ’ Real-World Stories’ section is also plotted in Fig 3, with the range [–1, –0] representing negative emotions and (0,1] representing positive emotions. As can be seen from the figure, the overall mood is biased towards positive, and the overall positivity is slightly lower than that of the ’Cultural China’ section, with the highest concentration being neutral to slightly positive. In the comments on the real-world story videos on YouTube (see Fig 4), the proportion of positive reviews (53.1%) was far greater than the negative ones (11.9%). Hence, it can be said that most people have a positive attitude toward these real-world story videos, which are well-received by most of the audience. Similarly, owing to the accuracy limitation of the sentiment-detection method, some sentences that contain negative emotional words but express positive or complex emotions are recognized as negative reviews. Therefore, compared with existing data, the actual number of positive comments should be even higher.

Both ’Cultural China’ and ’World Stories’ have a high proportion of neutral feelings, reaching 32.8% and 35% respectively. The peak number of comments appeared around the threshold, indicating that many comment sentiments were weak. This is related to ’Genshin Impact’ being a game, and there are many discussions or repetitions of in-game characters, plots, and dialogues in the comments section that do not contain the words included in the Textblob package, so it is very reasonable to express a large number of neutral judgments.

### (3) Emotional experience type: Cultural transmission under positive emotional experience

**Fig 5:**
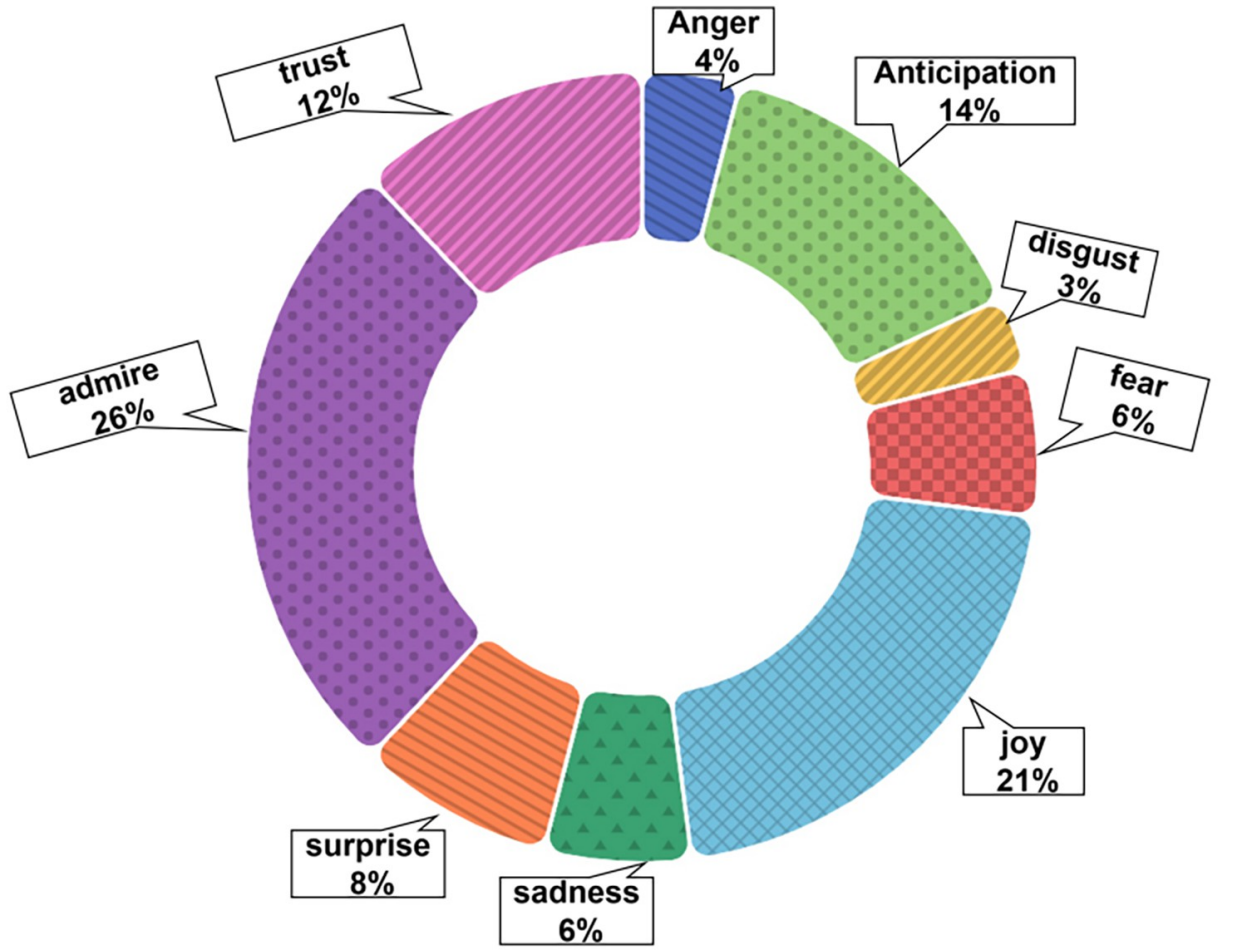
Proportion of nine major sentiment categories in partial comments with bias towards ’Cultural China’.

After adding the ’admire’ emotion type, 8,499 emotional expression comments were identified out of 12,682 comments in YouTube’s ’Cultural China’ section. The statistical results of the nine major types of emotions (see Fig 5) show that ’admire’ (26%), ’joy’ (21%), and ’anticipation’ (14%) account for the most positive emotions, but intense negative ’anger’ (4%), ’fear’ (6%), and ’disgust’ (3%) also have a certain proportion. Positive emotional experiences primarily focus on users expressing their love for the game and derivative videos, anticipating future storylines, and viewing favorite characters.

There is also a significant portion of the audience’s positive emotions focused on the appreciation for the Chinese culture in the game-making process and the hopes for more Cultural Chinese elements in future games, like the comment from user A: ’Props to them for hiring a professional Opera singer and sticking with their culture, hope to see more like this’. Strong negative emotions are mostly single words or phrases, such as ’it’s awful’, ’bad’, ’terrible’, etc. Others expressed dissatisfaction due to their favorite characters not appearing in the game or quotes from the storyline containing strong negative words.

**Fig 6:**
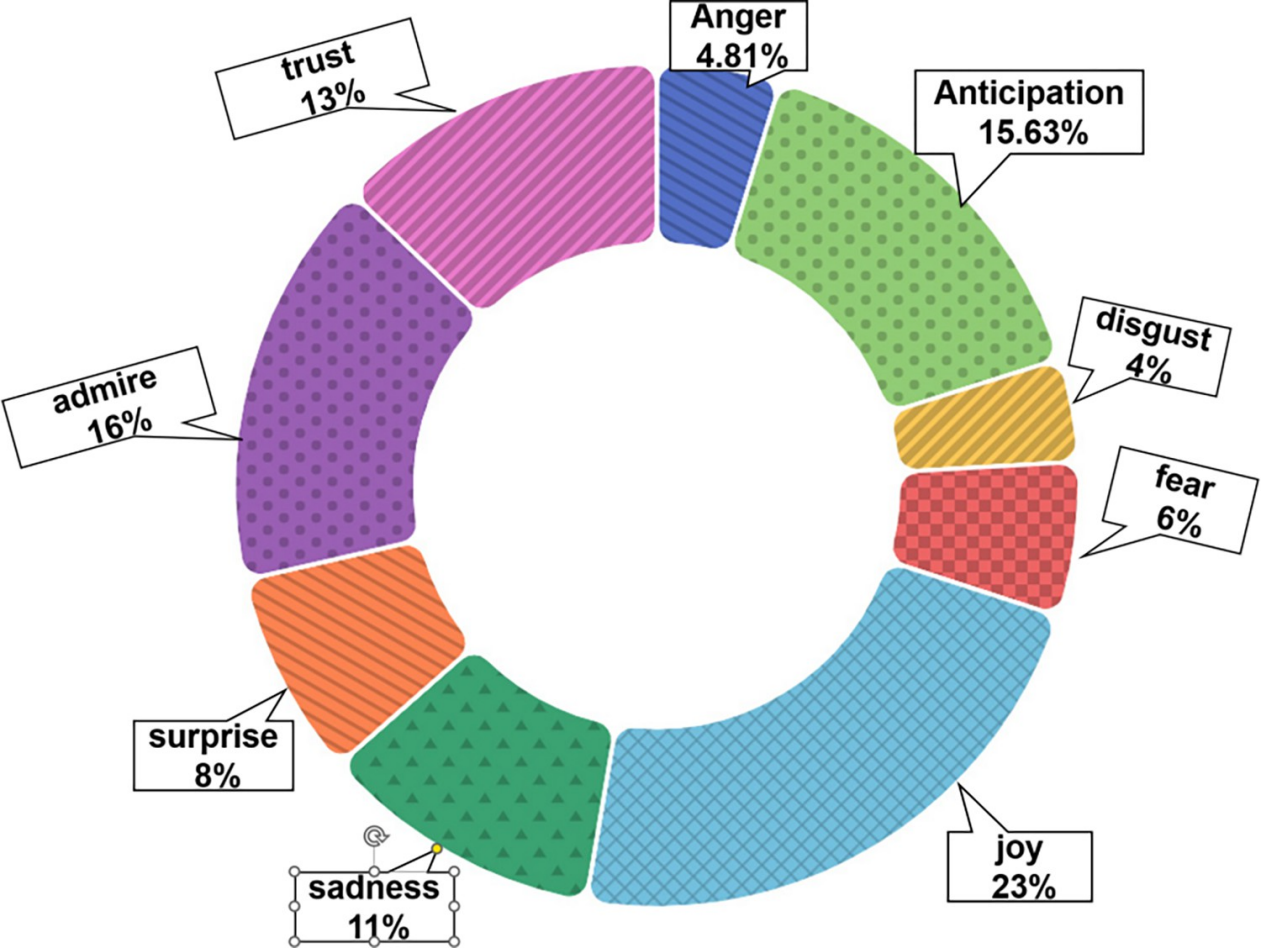
Proportion of nine major sentiment categories in partial comments with bias towards ’A Story of the World’.

After adding the emotion type of ’admire’ from the 9256 comments obtained in the ’World Story’ section on YouTube, 6388 sentiment expression comments were identified. The statistical results of the nine major categories of emotional comments show (see Fig 6) that positive emotional experiences such as ’admire’ (16%), ’joy’ (23%), and ’anticipation’ (15.63%) account for the majority, but more intense negative emotional experiences such as ’anger’ (4.81%), ’fear’ (6%), ’sadness’ (11%) also account for a certain proportion. These positive emotional experiences mainly concentrate on users’ admiration for the game scene design, love for the music style, etc., such as Commenter B: ’I cried for this cutscene. Absolutely beautiful and amazing. The visuals and music were so matching!’. Many of the audience’s positive emotions are concentrated on the cultural resonance of their cultural elements reproduced in Genshin Impact, such as Commenter C: ’Literally so happy that my culture is being represented in such a beautiful way’. On the other hand, strong negative emotions are expressed by repeating the plot’s original words with strong negative vocabulary and ’I cried for this cutscene’, which were categorized as ’sadness’.

Overall, the emotional experiences in the ’Cultural China’ and ’World Stories’ sections have a high proportion of positive emotions, indicating the audience has a high reputation and appreciation for Genshin Impact and its derivative videos. There is a high degree of recognition and cultural resonance regarding the cultural elements conveyed in the game. Among the negative emotional experiences, besides some phrases and vocabulary that cannot be deeply analyzed, many surface words express sadness but share love and positive emotions. This shows domestic and international players have positive overall emotional experiences with Genshin Impact and its derivative videos. Moreover, Genshin Impact effectively conveys cultural elements, allowing overseas players to recognize Chinese cultural aspects while resonating with their culture.

### (4) Semantic network analysis: Similarities and differences in the focal points of ’Cultural China’ and ’World Stories’

#### ①. Word Frequency Analysis Results

**Fig 7:**
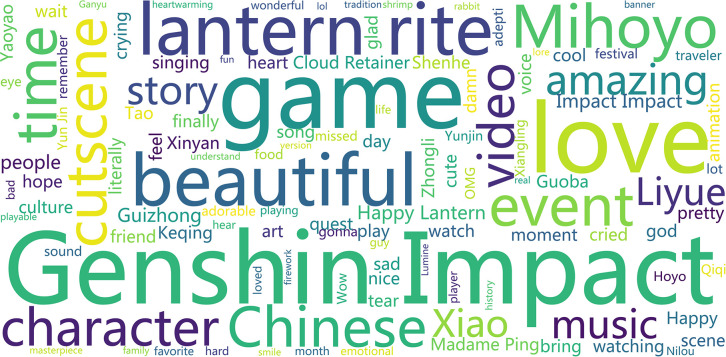
Word cloud of comments section with bias towards ’Cultural China’.

**Fig 8:**
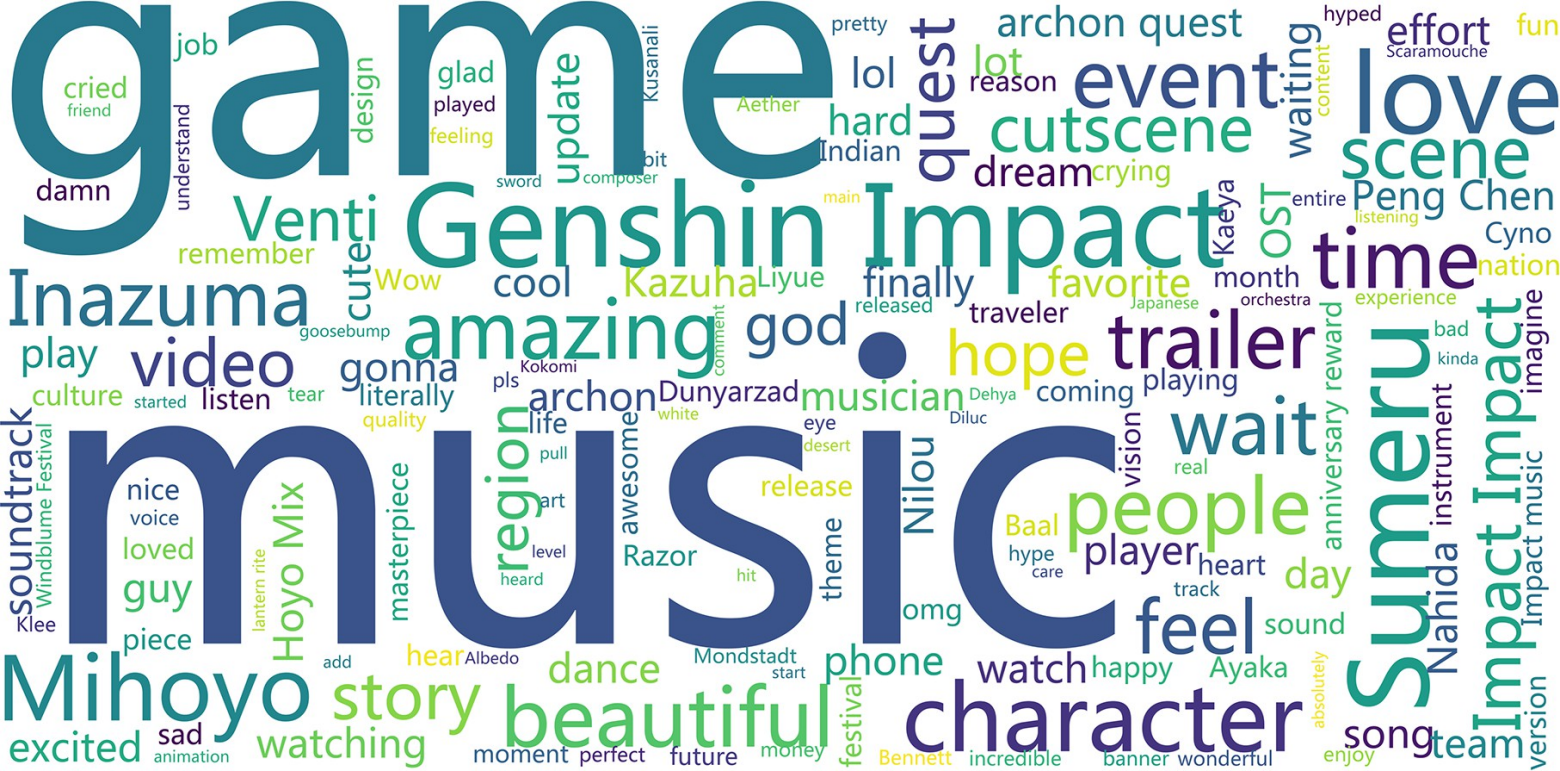
Word cloud of comments section with bias towards ’A Story of the World’.

To further parse the discussion content in the comment section and present the high-frequency vocabulary in the comment section more intuitively, the study visualized the word clouds of high-frequency words for the parts of ’Cultural China’ and ’World Stories’ acquired, respectively (see Figs 7 and 8).

The following features can be summarized from the ’Cultural China’ section:

1. A large number of words carrying Chinese cultural symbols have appeared, such as ’Happy Lantern’(’Lantern Rite’ is an annual ’Sea Lantern Festival’ in the game, an allusion to the Chinese New Year in reality), ’Chinese’, ’Chinese traditional’, ’Chinese opera’, ’Culture’. This shows that domestic and foreign players and audiences on YouTube have received Chinese elements in ’Genshin Impact’ very well and provided feedback.
2. Many laudatory words have appeared, such as ’Amazing’, ’Beautiful’, ’Pretty’, ’Awesome’, ’incredible’, and ’masterpiece’. This shows the praise and love for ’Genshin Impact’ and its derivative videos from domestic and foreign players and audiences on YouTube.
3. A large number of emotion-expressing words have appeared, such as ’Loved’, ’Cool’, ’Happy’, ’Crying’. This shows that ’Genshin Impact’ and its derivative videos can fulfill the emotional value of domestic and foreign players and audiences, bringing rich emotions.
4. As a game, the comment section also has a large number of names of characters or regions in the game, such as ’Liyue’ (an allusion to ancient Chinese place names), ’Zhongli’ (’Rock King Emperor’, an allusion to ancient Chinese gods), ’Yunjin’ (singer of Beijing Opera ’Goddess Splitting the Spectator’). This also reflects that domestic and foreign players and audiences are highly concerned and enthusiastic about regions with Chinese cultural characteristics and characters designed with unique Chinese features.

**Fig 9:**
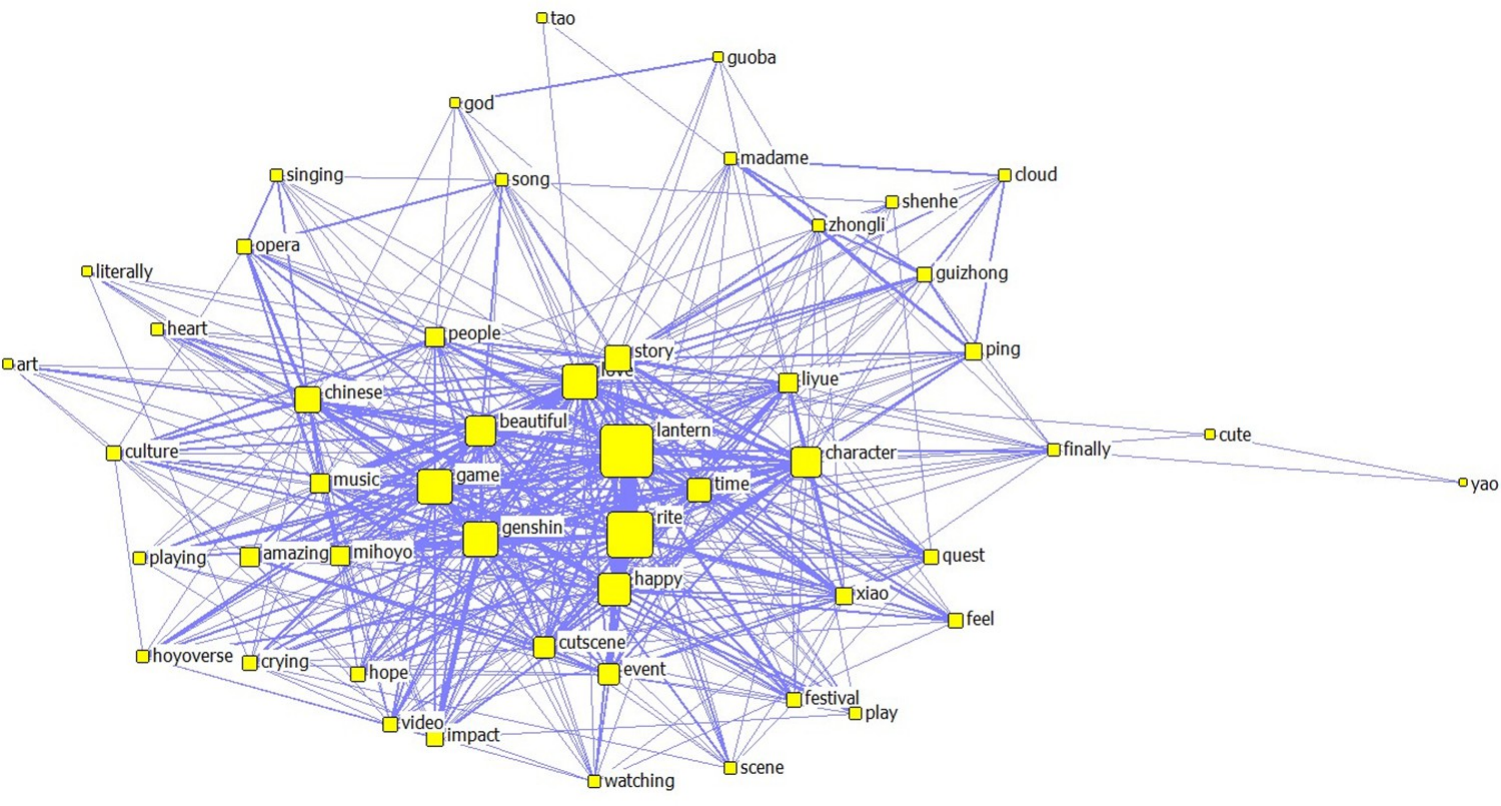
Co-occurrence network map of keywords in YouTube comments with bias towards ’Cultural China’.

**Fig 10:**
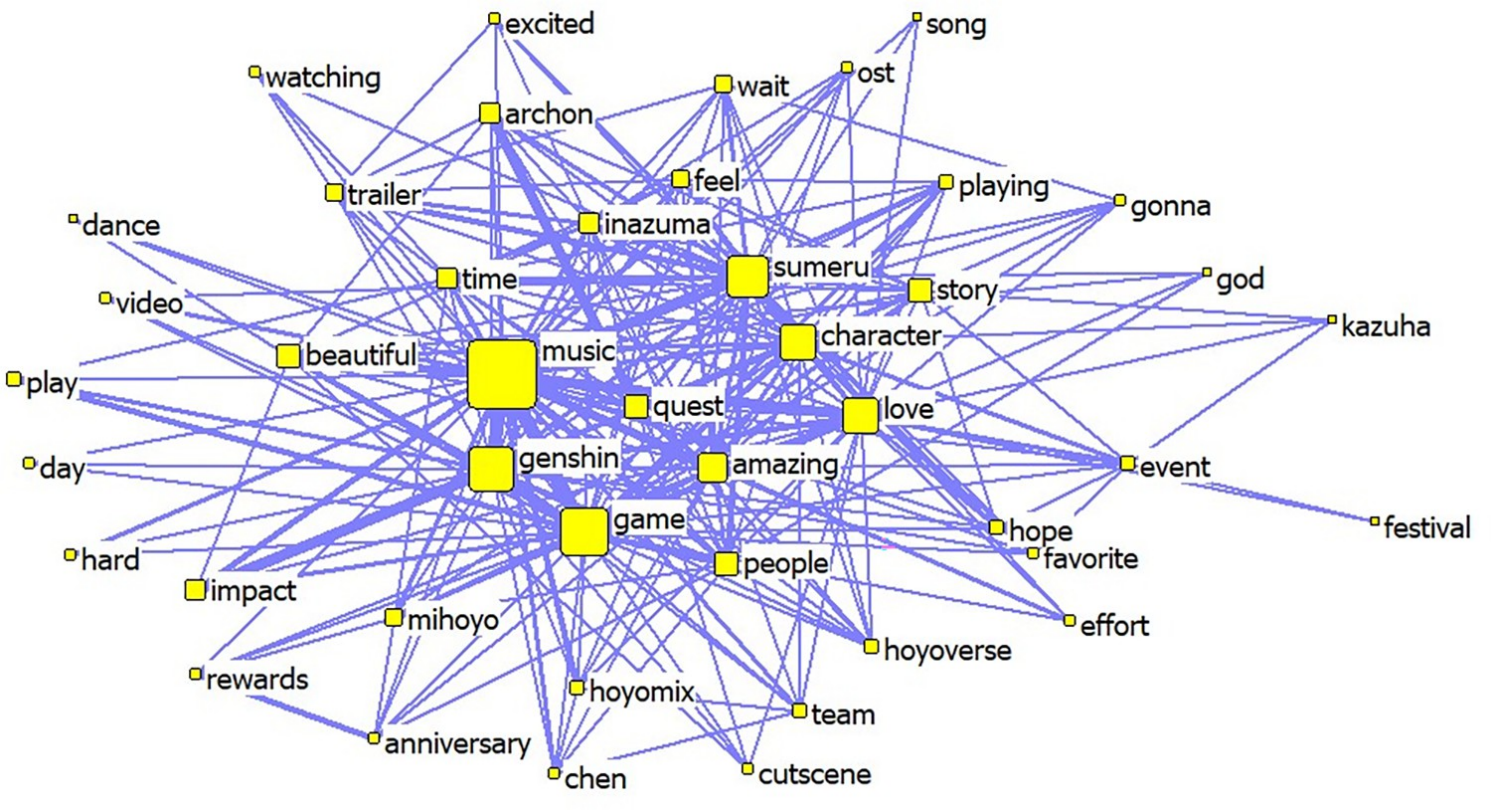
Co-occurrence network map of keywords in YouTube comments with bias towards ’A Story of the World’.

The following characteristics can be summarized from the ’World Stories’ section of the graph:

1. Unlike the ’Cultural China’ section, the ’World Stories’ section includes music-related content such as ’Music’, ’Instrument’, ’Listen’, ’OST’, and ’Orchestra’. As the ’ninth art’, games are a platform that utilizes the advantages of the eight major arts, including ’music’. ’Different styles of music are created by laboring people through long-term production and life practices and are closely related to the local natural environment, cultural traditions, and folk customs [19]. Therefore, it is questionable whether the carefully crafted musical pieces from various regions in ’Genshin Impact’ become the focus for domestic and foreign players and audiences on YouTube, especially foreign audiences, as clues to identify their oral traits;
2. Like the ’Cultural China’ section, there are numerous complimentary words and emotional expressions, reflecting the love and emotional value that domestic and foreign players and viewers on YouTube get from ’Genshin Impact’;
3. Like the ’Cultural China’ section, there are many games character names with costume characteristics from different regions of the world. At the same time, along with ’Inazuma’ (alluding to ancient Japan) and ’Sumeru’ (alluding to India, the Middle East, and the Arab regions), there are also high-frequency words like ’Japanese’, ’Chinese’, and ’Indian’. This shows that the cultural symbols conveyed by ’Genshin Impact’ have deeply affected domestic and foreign players and viewers, making them feel the cultural symbols of their respective countries or regions.
4. ②. The results of the co-word analysis indicate that the ’Cultural China’ section of the YouTube co-word network contains 50 nodes and 974 edges (see Figs 9 and 10), while the ’World Stories’ section of the YouTube co-word network contains 50 nodes and 538 edges. Generally, the content discussed and views expressed by the players and audience in the ’Cultural China’ section are more diversified and enriched. In contrast, the comments posted by players and audiences in the ’World Stories’ section were relatively more concentrated

From the perspective of the centrality of nodes, the ’Cultural China’ segment occupies important positions in the co-line network, including ’Lantern Rite’ (Spring Festival), ’Chinese’, ’Culture’, ’Luyue’ (ancient China), the deep imprint of Chinese culture, as well as ’Love’, ’Happy’, ’Amazing’ and other emotional expressions. These words have strong associations that can also affect the co-lines of other related terms. Additionally, ’Zhongli, Shenhe, Ping’, and other Chinese characters are important discussion topics for players and audiences. In the ’World Story’ section, the terms with high centrality are ’Music’, ’Genshin’, ’Character’, and emotional expressions similar to those in the ’Cultural China’ section.

Some believe that aspects of the music system have gradually adapted to the structure and functional features of the sensory and memory systems of learners and ’users’ over several generations [20]. ’Music’ does not appear in the ’Cultural China’ section but takes a large proportion in the ’World Story’ section. This might be associated with miHoYo, a game company based in China, meticulously studying and producing background music in collaboration with local orchestras that match the regional characteristics of overseas regions. This makes players and audiences abroad feel more immersive and resonate culturally when they hear localized music from different areas.

Therefore, this difference is observed. From the boldness of the edges of each node (the number of co-lines), much valuable information can be extracted. The more noticeable connections in the ’Cultural China’ section include ’Lantern-Beautiful-Chinese-Culture’ and ’Liyue-Character-Ping(character)Xiao(character)’; in the ’World Story’ section, more noticeable connections include ’Beautiful-Music-Amazing’, and ’Love-Story-Character-Sumeru (India and the Middle East)-Inazuma (Ancient Japan)’. It shows that overseas players and audiences have greatly different focus points and expressions towards ’Cultural China’ and ’World Story’. In the ’Cultural China’ section, overseas players and audiences are more focused on and curious about traditional Chinese culture and character design and portrayal with cultural symbols and characteristics.

However, in the ’World Story’ section, overseas players and audiences pay more attention to aspects such as music and stories that can invoke a sense of cultural resonance, seeking similarities and commonalities between their culture and the ’Genshin’ world.

#### ③. Cluster Analysis Results

**Fig 11:**
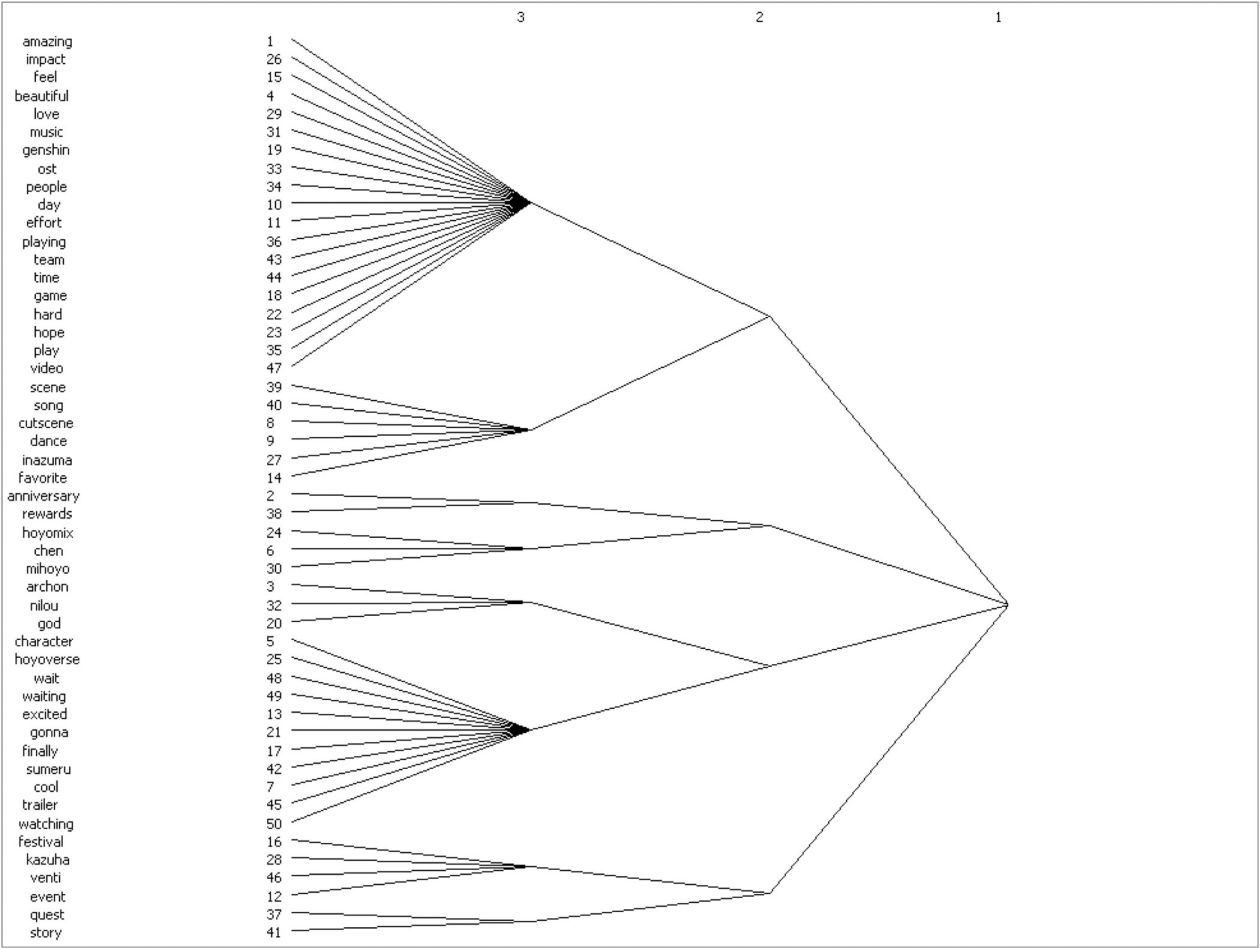
Dendrogram of comment clusters in partial comments with bias towards ’Cultural China’.

**Fig 12:**
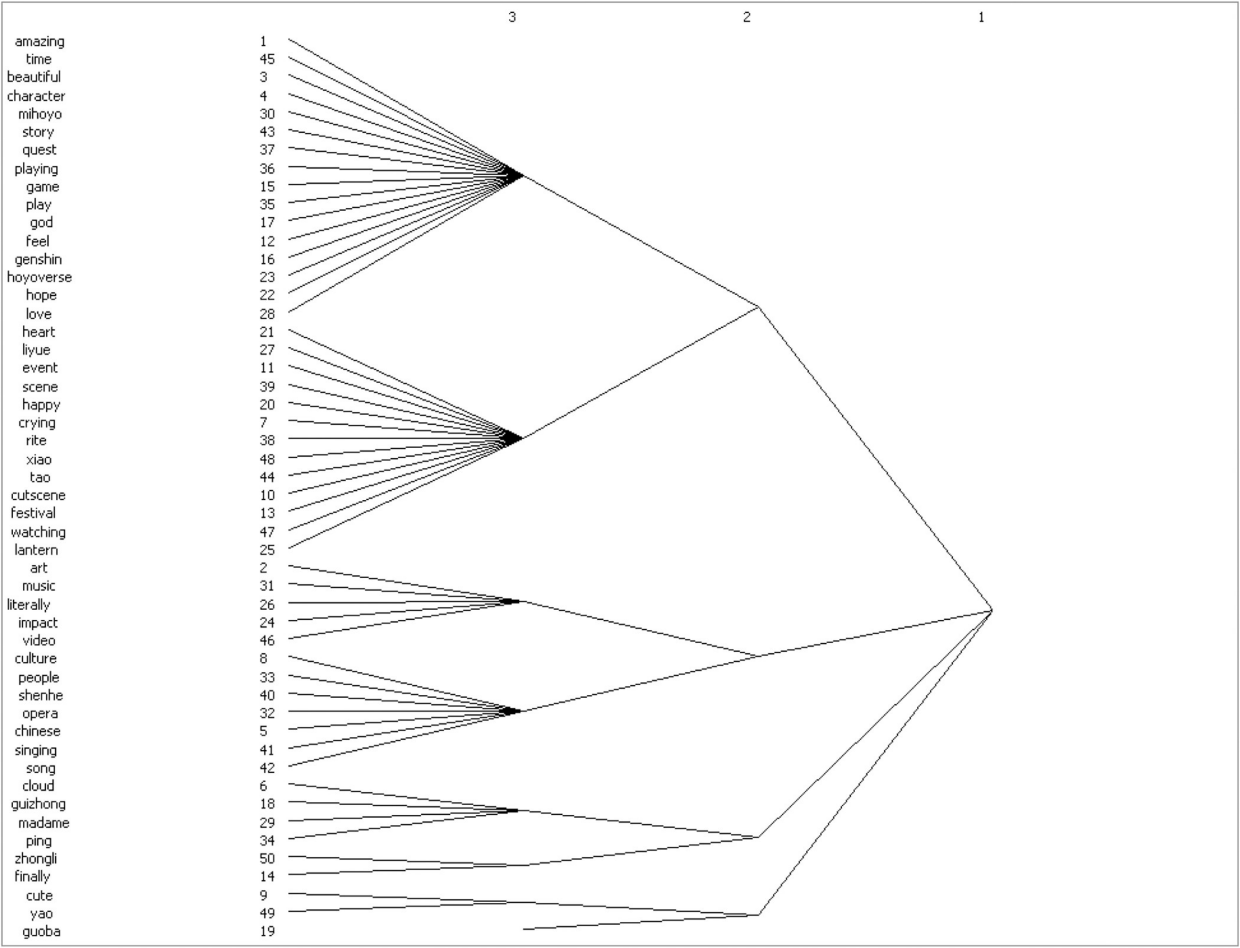
Dendrogram of comment clusters in partial comments with bias towards ’A Story of the World’.

After conducting modular analysis on the co-word matrix of high-frequency words in the YouTube comment section, both the ’Cultural China’ and ’World Stories’ parts obtained eight cohesive subgroups (see Figs 11 and 12).

The eight cohesive subgroups in ’Cultural China’ can be summarized and integrated into praises for Genshin Impact itself and game developer miHoYo; acclaims for the Chinese elements in Genshin Impact (scenes, festivals, character images, story plots), recognition of the communicative power of artistic symbols (music, text, videos) in Genshin Impact; praises for traditional Chinese culture, especially traditional drama (The Moonlight Seeker) with strong Chinese cultural symbols, and affections for the characters and related figures appearing in the Liyue (alluding to ancient China) scenes. The eight cohesive subgroups in ’World Stories’ can be summarized and integrated into praises for Genshin Impact itself and game developer miHoYo; high recognition of the cultural elements (music, dance, scenes) of the Inazuma (alluding to ancient Japan) region; affirmations of the exquisite background music conforming to local characteristics produced for each region and the production team; acclaims for the cultural elements (character design, scenes) of Sumeru (alluding to India and the Middle East); and loves for the story plots and periodic local festival events in the ’World Stories’ regions.

Thus, it can be seen that both the ’Cultural China’ and ’World Stories’ parts of Genshin Impact and its derivative videos are deeply loved by players and viewers, who also express their admiration for game developer miHoYo.

MiHoYo’s elaborate production of Genshin Impact was successful, integrating many Chinese and world cultural elements and symbols that have been well absorbed and recognized by players worldwide. In the cross-cultural communication of Genshin Impact, players can both experience ’exotic’ cultures and find cultural elements from their cultural circles, thus generating a sense of cultural resonance. At the same time, Genshin Impact uses background stories, game tasks, and derivative videos to successfully shape the images of characters and non-player characters (NPCs), making them subjects of extensive discussion. The cultural image-shaping of these characters serves as an excellent carrier.

It can also be seen that the meticulous work in the music of Genshin Impact is highly rewarding. The music in the Inazuma and Sumeru regions is well received, enhancing the sense of immersion in the game and giving overseas players a sense of intimacy and resonance. For the ’Cultural China’ part, domestic and overseas players and viewers are fascinated by The Moonlight Seeker (traditional Peking opera), triggering heated discussions and arousing players’ strong interest in traditional and Chinese culture.

## 6. Conclusion

Based on the objective YouTube user comment data, the cultural interactions in the cross-cultural communication of electronic games were examined from the perspective of diverse cultures, and the first four research questions were answered.

Through online text analysis, this study makes the following findings from the perspective of overseas players and viewers.

First, the sentiments of overseas players and viewers are mainly positive, but negative emotions also account for a certain proportion. Tracing back to the original texts reveals that the negative emotions mainly stem from not seeing their favorite characters, dissatisfaction with villain characters, repetition of original texts in the game, and so on, with occasional irrelevant negative remarks.

Second, the communication of Chinese and world cultures in Genshin Impact and its derivative videos provides viewers with good emotional experiences such as ’admire’, ’joy’, and ’anticipation’. The game was then recognized and confirmed. Players obtain positive emotional experiences of happiness and surprise during play and viewing, and expectations are generated for the subsequent plots and content.

Third, the main topics of ’Cultural China’ and ’World Stories’ and real-world stories have commonalities and differences. The difference is that ’Cultural China’ mainly focuses on Chinese-style festivals and items with strong Chinese cultural symbols in the game, such as the Peking opera and food. In contrast, real-world stories focus more on topics of in-game scenery and music that are more likely to evoke cultural resonance and search for cultural characteristic elements due to the overseas player and viewer sample selected.

Fourth, in the cross-cultural communication of Genshin Impact and its derivative videos, overseas players can find cultural elements in their cultural circles in the game, such as scenes, music styles, and game settings, thus generating a sense of cultural resonance or even further associative thinking. One Latin American player commented on the Sumeru OST ’Forest of Jnana and Vidya’ Preview MV:

This is what true globalization and cultural exchange is all about. Bringing together the quintessence of various cultures, this has become one of the greatest works of modern art. Music is a universal language; it transcends hatred and division, brings people together, and unites them in harmony with peace and love. Whether it is Indian music, South Asian music, West Asian music, Arabic music, etc. does not matter. In addition, it shows the Chinese understanding of things beyond the Silk Route. Mihoyo has taken the art of representation to a new level; it is pure art and respects thousands of years of culture through generations of peaceful or violent exchange. I’m a Latin American raised amid constant squabbling between fellow Latin Americans; we are all one whole, stop squabbling and enjoy art [21].

This research objectively proves through data that the exquisite and meticulous production of Genshin Impact and derivative videos successfully promotes traditional Chinese culture by incorporating cultural symbols from countries and regions worldwide into scenes, music, character designs, stories, and other aspects under its fictional worldview. While allowing domestic and overseas players to experience exotic cultural colors, they can also feel the cultural symbols of their cultural circles in the game and generate cultural resonance.

Through the ’ninth art’, Genshin Impact and its derivative videos successfully tell the stories of ’Cultural China’ and the ’World’ and provide players and viewers with excellent emotional experiences and emotional value. As the game continues to update the maps, it is believed that the future opening of Danfeng, Natlan, and Snezhnaya will also bring more surprises and allow more players to feel the cultural elements of different regions in the game so that more players from more regions can find cultural aspects from their cultural circles.

The discussion on cultural interactions in the cross-cultural communication of ’the ninth art’ also provides new perspectives for promoting Chinese culture overseas and cross-cultural communication, with constructive significance for implementing the national strategy of ’telling Chinese stories well and spreading Chinese voice.

## 7. Discussion

Based on the above conclusions, this research provides the following insights into how to better use the ’ninth art’ for cross-cultural communication of Chinese stories:

First, do not dump large amounts of cultural elements and symbols, but utilize the high immersion and multiple art forms of games, on the premise of prioritizing game quality, to slowly express the intended cultural connotations through scenes, music, plots, and let players and viewers slowly feel, accept, and absorb in immersive experiences, making them more sustainable.

Second, the cultures of different countries and regions have coexisting similarities and regionalities, and players’ and viewers’ cognitions differ; however, emotional experiences have interoperability. The creation of the ’ninth art’ is based on universal human emotions and values, and emotional experiences will more easily break through cultural barriers. Therefore, cultural elements and information should encompass more diverse perspectives, as in the production of Genshin Impact, using excellent works of art to provide players with emotional experiences of ’being one community with a shared future’.

Third, resonance in musical empathy is valued in terms of social bonding. Horizontal crystallization of social consensus in weak relational scenes highlights the value of music as a medium, in addition to its content value [22]. Therefore, when using the ’ninth art’ to spread culture, the shaping of music must be emphasized through in-depth investigation and research on musical elements from countries and regions integrated into games. At the same time, the innovation of traditional culture must be embraced, as exemplified by The Moonlight Seeker in Genshin Impact, which triggered enthusiastic discussions and various language adaptations among domestic and overseas players and viewers. Production should emphasize music as a medium for cultural communication.

Fourth, the perspective of ’cultural centralism’ should be abandoned. While exporting culture through the ’ninth art’, differences with other cultures must also be respected. While displaying China’s cultural heritage, accumulated over thousands of years, alien cultures must be absorbed and accepted, making works more inclusive and demonstrating China’s image as a great power. An in-depth investigation is needed concerning cultural origins when producing content about other cultures. Gaining full respect from overseas players while bringing them cultural resonance will make them more willing to absorb the content and traditional Chinese cultural elements that the game seeks to express to achieve better cross-cultural communication effects.
